# Efficacité et tolérance des antiviraux à action directe dans le traitement de l'hépatite C au CHU Joseph Raseta Befelatanana d'Antananarivo, Madagascar

**DOI:** 10.48327/mtsibulletin.2021.153

**Published:** 2021-09-16

**Authors:** C.I. Razafindrazoto, A.S. Rasolonjatovo, N.H. Randriamifidy, S.S. Rabarioely, A.L.R. Rakotozafindrabe, T.H. Rabenjanahary, S.H. Razafimahefa, R.M. Ramanampamonjy

**Affiliations:** 1 Unité de soins, de formation et de recherche, hépato-gastro-entérologie, Antananarivo, Madagascar. Faculté de médecine d'Antananarivo, Université d'Antananarivo, Madagascar; 2 Unité de soins, de formation et de recherche, hépato-gastro-entérologie et de médecine interne, Fianarantsoa, Madagascar. Faculté de médecine de Fianarantsoa, Université de Fianarantsoa, Madagascar

**Keywords:** Hépatite virale C, Antiviraux à action directe, Hôpital, Antananarivo, Madagascar, Océan Indien, Viral hepatitis C, Direct-acting antiviral drugs, Hospital, Antananarivo, Madagascar, Indian Ocean

## Abstract

**Objectif:**

Notre travail avait pour but d’évaluer l'efficacité et la tolérance des antiviraux à action directe dans le traitement de l'hépatite C à Madagascar.

**Méthodologie:**

Il s'agit d'une étude rétrospective réalisée, de mars 2018 à février 2020, dans le service d'hépato-gastro-entérologie du Centre hospitalier universitaire Joseph Raseta de Befelatanana.

**Résultats:**

Au total, 35 patients ont été inclus, dont 24 ont reçu du sofosbuvir/ledipasvir ± ribavirine, 10 du sofosbuvir/ribavirine et 1 du sofosbuvir/velpatasvir. Trente-trois patients étaient naïfs de traitement et 2 patients avaient été initialement traités par l'association sofosbuvir/ledipasvir. La réponse virologique soutenue était de 94% (33/35) dans la population globale, chez 23 des 25 patients cirrhotiques et chez 10 sur 10 des non cirrhotiques. La réponse virologique soutenue était de 22 sur 24 pour l'association sofosbuvir/ledipasvir ± ribavirine, 10 sur 10 pour sofosbuvir/ribavirine et un sur un pour sofosbuvir/velpatasvir. Des effets indésirables, principalement insomnie et asthénie, ont été observés chez 13 patients.

**Discussion:**

Le faible effectif étudié et les contraintes financières des patients constituent les principales limites de notre étude.

**Conclusion:**

Les antiviraux à action directe sont efficaces et bien tolérés dans ce groupe de patients malgaches atteints d'hépatite C.

## Abstract (en)

**Objective:**

Our work aimed to assess the efficacy and safety of direct-acting antiviral drugs in the treatment of hepatitis C in Madagascar.

**Methods:**

This retrospective clinical study was carried out from March 2018 to February 2020 in the hepato-gastro-enterology department of the University Hospital Center Joseph Raseta de Befelatanana.

**Results:**

A total of 35 patients were included, out of which 24 received sofosbuvir/ledipasvir ± ribavirin, 10 sofosbuvir/ribavirin and one sofosbuvir/velpatasvir. Thirty-three patients were naïve to the treatment and 2 patients were initially treated with the sofosbuvir/ledipasvir combination. The sustained virologic response was 94% (33/35) in the general population, 23/25 in cirrhotic patients and 10/10 in non-cirrhotic patients. The sustained virologic response was 22/24 for sofosbuvir/ledipasvir ± ribavirin, 10/10 for sofosbuvir/ribavirin and 1/1 for sofosbuvir/velpatasvir. Adverse effects were observed in 13 patients, mainly asthenia and insomnia.

**Discussion:**

The small number of patients with hepatitis C treatments and their limited financial resources are the main limits of this survey.

**Conclusion:**

Direct-acting antivirals are effective and characterized by good tolerance in these Malagasy hepatitis C patients.

## Introduction

L'hépatite C constitue un problème de santé publique dans les pays à faible revenu [21]. La situation de l'hépatite C est peu documentée à Madagascar. L'estimation de la prévalence (1,2%) de l'infection par le virus de l'hépatite C (VHC) en population générale reste difficile à apprécier, notamment du fait de la non-représentativité des échantillons de populations étudiées [25]. Les VHC génotype 1 (GT-1) et 2 (GT-2) prédominent à Madagascar [16]. Les recommandations actuelles préconisent de traiter tous les patients porteurs du virus de l'hépatite C, en vue d'une éradication de l'infection d'ici 2025 à 2030 selon la politique de chaque pays. L'utilisation des antiviraux à action directs (AAD) a révolutionné la prise en charge de l'hépatite C chronique [7].

Dans notre pratique quotidienne, la prise en charge de l'hépatite C reste problématique devant la quasi-impossibilité des patients à réaliser les prélèvements virologiques et de bénéficier du traitement, du fait des coûts très élevés, au-dessus du pouvoir d'achat de la population malgache en général.

Notre travail a pour but d’évaluer l'efficacité et la tolérance des antiviraux à action directe dans le traitement d'hépatite C à Madagascar.

## Patients et Méthodes

Il s'agit d'une étude analytique, rétrospective, réalisée à Antananarivo (Madagascar) sur une période de 2 ans (1^er^ mars 2018 au 28 février 2020). La population d’étude était constituée par les patients atteints de VHC suivis dans le service d'hépato-gastro-entérologie au Centre hospitalier universitaire Joseph Raseta Befelatanana. Ce CHU est un hôpital public universitaire traitant les pathologies de médecine interne. Le service de gastro-entérologie assure la prise en charge de tous les patients atteints de maladies aiguës et chroniques du foie, du pancréas et du tube digestif y compris le dépistage des cancers digestifs. Le service comporte 27 lits d'hospitalisation conventionnelle et 2 unités fonctionnelles dont une unité d'hépato-gastro-entérologie générale et une unité d'endoscopie. Le recrutement des patients se fait de manière générale via les services des urgences.

Ont été inclus les patients avec des anticorps anti-VHC positifs, virémiques, naïfs ou rechuteurs ou non répondeurs, tous génotypes confondus, cirrhotiques ou non, en absence de carcinome hépatocellulaire, mis sous combinaison sofosbuvir et autres antiviraux à action directe. Les patients perdus de vus et sans surveillance de la charge virale ont été exclus.

Durant la période d’étude, nous avons recensé 57 patients atteints de VHC (22 patients en hospitalisation et 35 en consultation externes). Seuls les 35 patients vus en consultation externe, recrutés de façon consécutive, ont pu bénéficier de traitement. Aucun patient n'a été perdu de vue durant la période d’étude. Les AAD étaient inaccessibles pour les patients hospitalisés du fait de leurs niveau socioéconomique faible. Les AAD ont été suggérés à tous les patients atteints de VHC à la fin de l'hospitalisation ou lors des consultations externes. Au final, seuls les patients ayant le moyen d'en acheter en ont bénéficié.

Les paramètres étudiés étaient l’âge, le genre, le génotype (GT), le degré de fibrose, la présence ou non de cirrhose, la charge virale (ARN viral) à la 4^e^, 12^e^ et 24^e^ semaines de traitement, les effets indésirables. Le degré de fibrose était évalué par des tests non-invasifs, classé en 4 stades de sévérité croissante, F0 correspond à une absence de fibrose et F4 correspond à la cirrhose constituée. Le diagnostic de cirrhose était basé sur les résultats de l'exploration hépatique non invasive: clinique, échographie hépatobiliaire et fibrotest.

Le génotype et l'ARN VHC ont été réalisés par PCR en temps réel (RT-PCR reverse-hybridation LiPA et RT-PCR Cobas 8800 Roches, Cerba, France), avec une limite inférieure de quantification de 10 UI/ml.

Les différents protocoles ont été suggérés selon le génotype du VHC, le degré de fibrose, la disponibilité des molécules et l'accessibilité financière des patients:

- le traitement par sofosbuvir 400 mg et velpatasvir 100 mg pour un comprimé par jour pendant 12 semaines a été proposé à tous les patients atteints du VHC quel que soit le génotype. Peu de patients pouvaient en bénéficier du fait du coût;
- les patients atteints de VHC (génotypes 1, 2, 3 et 4) non cirrhotiques et cirrhotiques compensés (CHILD-PUGH A) qui ne pouvaient pas bénéficier du sofosbuvir/velpatasvir ont été mis sous sofosbuvir 400 mg et ledipasvir 90 mg par comprimé par jour. Les patients avec cirrhose décompensée (CHILD-PUGH B et C) ont bénéficié du sofosbuvir 400 mg et du ledipasvir 90 mg par comprimé par jour associé à la ribavirine 1 000 mg/j (poids < 75 kg) ou 1 200 mg/j (poids > 75 kg) 2 fois par jour pendant 12 semaines;
- les patients atteints de VHC de génotypes 1, 2, 3 et 4 cirrhotiques ou non cirrhotiques qui ne pouvaient pas bénéficier du sofosbuvir/velpatasvir et du sofosbuvir/ledipasvir ± ribavirine étaient mis sous combinaison de sofosbuvir 400 mg par comprimé par jour et de ribavirine 1 000 mg/j (poids < 75 kg) ou 1 200 mg/j (poids > 75 kg) 2 fois par jour pendant 12 semaines.

La durée du traitement de tous les malades était de 12 semaines quel que soit les schémas utilisés. Nous avons suivi les patients jusqu’à 12 semaines après l'arrêt du traitement afin d’évaluer l'efficacité du protocole thérapeutique et sa tolérance avec un suivi régulier de la charge virale. La charge virale était mesurée avant le début du traitement, puis à la 4^e^ semaine afin d’évaluer la réponse virologique rapide (RVR) et à la 12^e^ semaine de traitement pour l’évaluation de la réponse en fin de traitement (RFT). La dernière mesure de la charge virale était réalisée 12 semaines après l'arrêt du traitement afin d’évaluer la réponse virologique soutenue (RVS) correspondant au critère de jugement principal de cette étude et synonyme de guérison.

La tolérance était évaluée en recueillant tous les événements indésirables rapportés par le patient ou observés lors de la période du traitement et du suivi.

## Résultats

### Description globale de la population d’étude

Au total, 35 patients ont été inclus dont 22 femmes et 13 hommes. L’âge médian était de 61 ans (extrêmes: 34 à 76 ans). Vingt-cinq patients était cirrhotiques et 10 non cirrhotiques. Les génotypes 1, 2, 3 et 4 ont été retrouvés avec un taux respectif de 34%, 51%, 9%, 6%.

**Figure 1:**
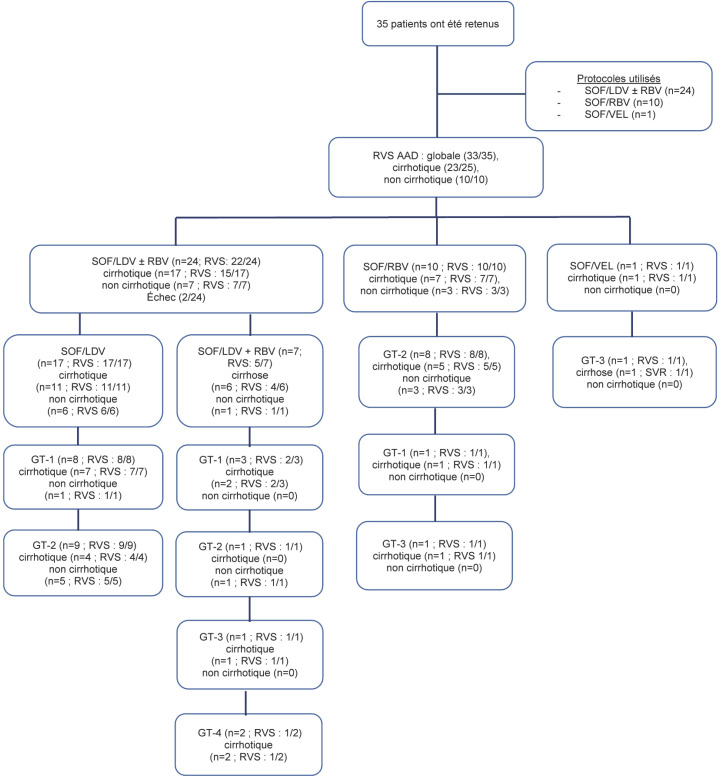
Flow-chart de nos patients Flow-chart of our patients

Vingt-quatre patients ont reçu du sofosbuvir/ledipasvir ± ribavirine, 10 du sofosbuvir/ribavirine et un du sofosbuvir/velpatasvir (Fig. 1).

Trente-trois patients étaient naïfs de traitement et deux patients avaient été initialement traités par l'association sofosbuvir/ledipasvir pendant 12 semaines. La fibrose sévère et la non-adjonction initiale de ribavirine constituaient probablement les principaux facteurs d’échec pour ces deux patients. Ces patients ont bénéficié en deuxième ligne du sofosbuvir/ledipasvir + ribavirine pendant 12 semaines. Les caractéristiques démographiques de notre population d’étude sont représentées dans le tableau I.

**Tableau I:** Caractéristiques démographiques et cliniques de la population d’étude Demographic and clinical characteristics of the study population

Variables	Effectifs
**Femme**	22
**Homme**	13
**Âge médian en années (extrêmes)**	61 (34 – 76)
**Génotypes**	
1b	12
2	6
2a/2c	9
2b	3
3	3
4	2
**Fibroactitest**	
F0 – F3	10
F4	25
**Cirrhose**	25
compensée	16
décompensée	9
**Charge virale (UI/ml)**	
≤ 500 000	6
≥ 500 000	29
**Statuts thérapeutiques antérieurs**	
naïfs	33
rechuteurs	2
**Protocoles thérapeutique utilisés**	
SOF+LDV+RBV	24
SOF+RBV	10
SOF+VEL	1

SOF+LDV±RBV = sofosbuvir + ledipasvir ±ribavirine; SOF+RBV = sofosbuvir + ribavirine; SOF+VEL= sofosbuvir + velpatasvir

### Efficacité des antiviraux à action directes

La RVR et la RFT étaient respectivement de 80% (28/35) et 100% (35/35). Tous nos malades ont effectué le dosage de l'ARN viral 12 semaines après l'arrêt du traitement. Trente-trois patients (94%) avaient une réponse virologique soutenue (RVS). La RVS était de 92% (23/25) chez les patients cirrhotiques et de 10/10 chez les non-cirrhotiques. Elle était de 11/12 chez les GT-1, 18/18 chez les GT-2, 3/3 chez les GT-3, 1/2 chez les GT-4. La RVS selon les molécules était de 22/24 sous sofosbuvir/ledipasvir ± ribavirine, 10/10 sous sofosbuvir/ribavirine et 1/1 sofosbuvir/velpatasvir (Tableau II). Une rechute a été observée chez deux patients cirrhotiques, de génotype 1b et 4, sous sofosbuvir/ledipasvir + ribavirine.

**Tableau II:** Réponse virologique à la 4^e^ (RVR), 12^e^ (RFT) semaines du traitement et RVS (12 semaines après le traitement) selon les molécules utilisées Virological response at the 4th (RVR), 12^th^ (RFT) weeks of treatment and RVS (12 weeks after treatment) depending on the molecules used

Molécules	Génotypes (n)	RVR n/N	RFT n/N	RVS n/N	Échec n/N	p value
**SOF+LDV±RBV (N=24)**	1 (11) / 2 (10) / 3 (1) / 4 (2)	19/24	24/24	22/24	2/24	NS
**SOF+VEL (N=1)**	3 (1)	1/1	1/1	1/1	0/1	
**SOF+RBV (N=10)**	1 (1) / 2 (8) / 3(1)	8/10	10/10	10/10	0/10	

SOF: sofosbuvir; LDV: ledipasvir; RBV: ribavirine; VEL: velpatasvir; RVR: réponse virologique rapide; RFT: réponse virologique en fin de traitement; RVS: réponse virologique soutenue

### Tolérance et effets indésirables

Les AAD ont été bien tolérés chez 22 patients (63%). Aucun patient n'a présenté d'effet indésirable sévère nécessitant un arrêt de traitement. L'asthénie et l'insomnie étaient les deux principaux événements indésirables retrouvés chez les 13 patients ayant eu des effets indésirables.

## Discussion

L'arrivée des antiviraux à action directe (AAD) en 2011 a rendu l’élimination de l'hépatite C envisageable [3]. Ces antiviraux restent actuellement peu accessibles pour la population malgache du fait de leur coût. À Madagascar, les coûts des 12 semaines de traitement pour le sofosbuvir/velpatasvir, sofosbuvir/ledipasvir + ribavirine et sofosbuvir/ribavirine étaient respectivement de 820 €, 764 € et 550 €. Les publications sur le sujet sont rares en Afrique subsaharienne du fait des difficultés à faire des examens complémentaires (génotypes, charge virale) et de la non-accessibilité des AAD. Cependant, de nombreuses études ont démontré l'efficacité et l'excellente tolérance des AAD [1^1^, ^10^, ^11^, ^12^, ^15^, ^26^]. Notre travail est une première sur l’évaluation de l'efficacité et de la tolérance des AAD sur un échantillon de population malgache atteint d'hépatite C.

La RVR et la RFT de nos patients étaient élevées respectivement de 80% et 100%, comparables aux résultats de Mairamou Hamadou et al en 2018 au Cameroun (RVR et RFT: 73% et 99%) et proches de ceux de Sharafi et al en 2020 en Iran (RVR et RFT: 100%) [13, 18]. RVR et RFT ne sont pas forcément nécessaires pour évaluer l'efficacité des AAD. Afin de réduire le coût (coût d'un dosage de la charge virale: 64 €) et de simplifier le suivi des patients sous AAD à Madagascar, nous préconisons seulement deux dosages de la charge virale, avant l'initiation de traitement et 12 semaines après l'arrêt du traitement.

Dans l'ensemble, la RVS sous AAD était élevée dans notre population (> 90%). Il faut préciser que dans cette série, le VHC génotype 2 est majoritaire, expliquant à lui seul ces

## Résultats

Compte tenu du coût des traitements, il parait pertinent de génotyper les patients. Les taux de RVS dans les études varient de 71 à 100% [1, 3, 5, 8, 10, 11, 12, 13, 15, 18, 20, 22, 24, 26]. Notre taux de RVS était plus élevé que ceux dans les autres études sur une population sub-saharienne: au Cameroun (85%), Rwanda (87%) et sur des immigrés sub-sahariens aux USA (88%) et similaire à ceux en Afrique du Sud (96%) [8, 13, 20, 22].

Plusieurs études ont montré que la cirrhose constituait un facteur prédictif d'un échec de traitement sous sofosbuvir/ledipasvir ± ribavirine [2, 9, 14], pouvant expliquer cette rechute chez nos 2 patients cirrhotiques, de génotype 1 et 4, sous sofosbuvir/ledipasvir ± ribavirine. Sous réserve du faible effectif, le sofosbuvir/ribavirine dans notre étude avait une bonne RVS. Le coût du sofosbuvir/ribavirine est plus faible que celui des autres associations. Cette association pourrait être recommandée en première ligne chez les patients génotype 2 à Madagascar.

Actuellement, des traitements pangénotypiques sont de plus en plus utilisés dans la politique d’éradication de l'hépatite C dans de nombreux pays. La combinaison sofosbuvir/velpatasvir est la plus utilisée [7]. Deux études sur sofosbuvir/velpatasvir dans le traitement du VHC de tous génotypes existants (GT-1, 2, 3, 4, 5 et 6) ont affirmé sa supériorité avec des RVS variant de 95 à 100% [4, 6]. L'inconvénient de ce schéma thérapeutique réside dans son coût élevé. La majorité de la population malgache ne peut en bénéficier.

Des effets indésirables ont été observés chez 13 patients dans notre série. Aucun effet indésirable sévère nécessitant un arrêt de traitement n'a été rapporté. Nos résultats corroborent la majorité des résultats des études menées sur la thématique, montrant une excellente tolérance des AAD. Les effets indésirables tels que nausée, asthénie, céphalée, insomnie, prurit sont les plus rapportés [1, 2, 3, 4, 5, 6, 8, 9, 10, 11, 12, 13, 14, 15, 17, 18, 19, 20, 21, 22, 23, 24, 26]. Alors que l'anémie est l'effet indésirable le plus cité chez les patients sous ribavirine [15, 17, 23], aucun patient de notre série ayant reçu de la ribavirine n'a présenté d'anémie.

Le faible effectif étudié et les contraintes financières des patients constituent les principales limites de notre étude. Nos résultats montrent cependant que les antiviraux à action directe sont efficaces avec une excellente tolérance dans ce groupe de population malgache atteint d'hépatite C.

## Conclusion

L'hépatite C constitue un problème majeur de santé publique à Madagascar. L'arrivée des antiviraux à action directe en 2011 a rendu l’élimination de l'hépatite C envisageable. Les antiviraux à action directe sont efficaces et caractérisés par une bonne tolérance dans notre groupe de patients. Compte tenu de la prévalence majoritaire du VHC génotypes 1 et 2, du coût des AAD et des marqueurs viraux à Madagascar, nous recommandons l'utilisation du sofosbuvir/ledipasvir ± ribavirine et préconisons seulement deux dosages de la charge virale, avant le début du traitement et 12 semaines après son arrêt. Une étude sur un plus grand échantillon de population malgache est cependant souhaitable pour confirmer nos résultats.

## Remerciements

Les auteurs tiennent à remercier chaleureusement les membres du service de gastro-entérologie du CHU Joseph Raseta Befelatanana. Cette recherche n'a reçu aucune subvention spécifique d'agences de financement des secteurs publics, commerciaux ou à but non lucratif.

## Conflits D'intérêts

L'auteur déclare ne pas avoir de conflit d'intérêt.
